# Supplementary material for: Stochastic model of vesicular stomatitis virus replication reveals mutational effects on virion production
Source: PLoS Comput Biol. 2024 Feb 7;20(2):e1011373. doi: 10.1371/journal.pcbi.1011373 (PMC10878530; doi:10.1371/journal.pcbi.1011373)
Supplement: S1 Fig — These two graphs show how the time to load each model (B) and simulate the model (A) increase drastically as the number of reactions in the model increases. (PDF) [file pcbi.1011373.s001.pdf]

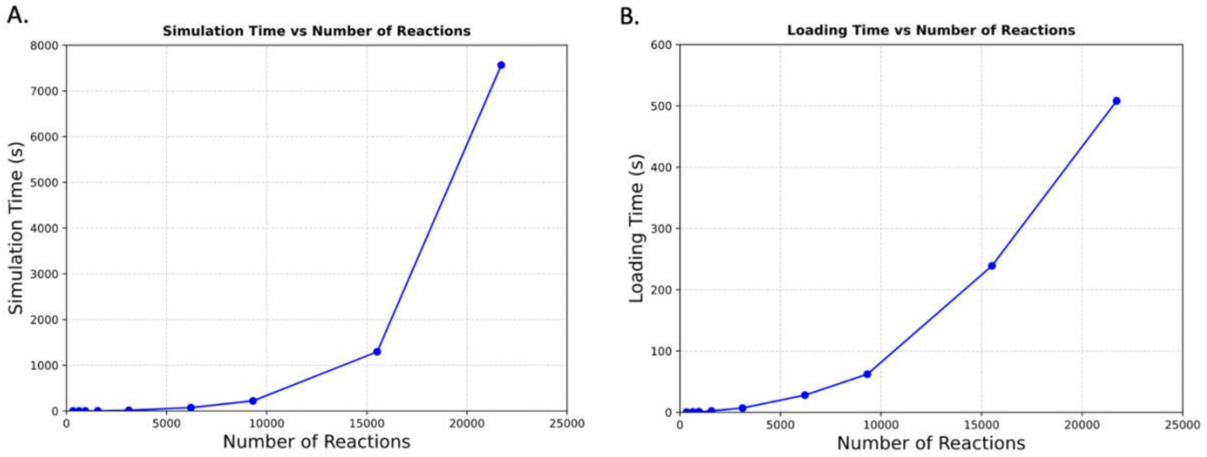

S1 Fig. Simulation and Loading time of ODE Models Increases as the Number of Reactions in the Model Increase.
