# Supplementary material for: Stochastic model of vesicular stomatitis virus replication reveals mutational effects on virion production
Source: PLoS Comput Biol. 2024 Feb 7;20(2):e1011373. doi: 10.1371/journal.pcbi.1011373 (PMC10878530; doi:10.1371/journal.pcbi.1011373)
Supplement: S2 Fig — These graphs show the distribution for the final number of virions produced for 10,000 simulations of each gene-shuffled variant in the training set. (PDF) [file pcbi.1011373.s002.pdf]

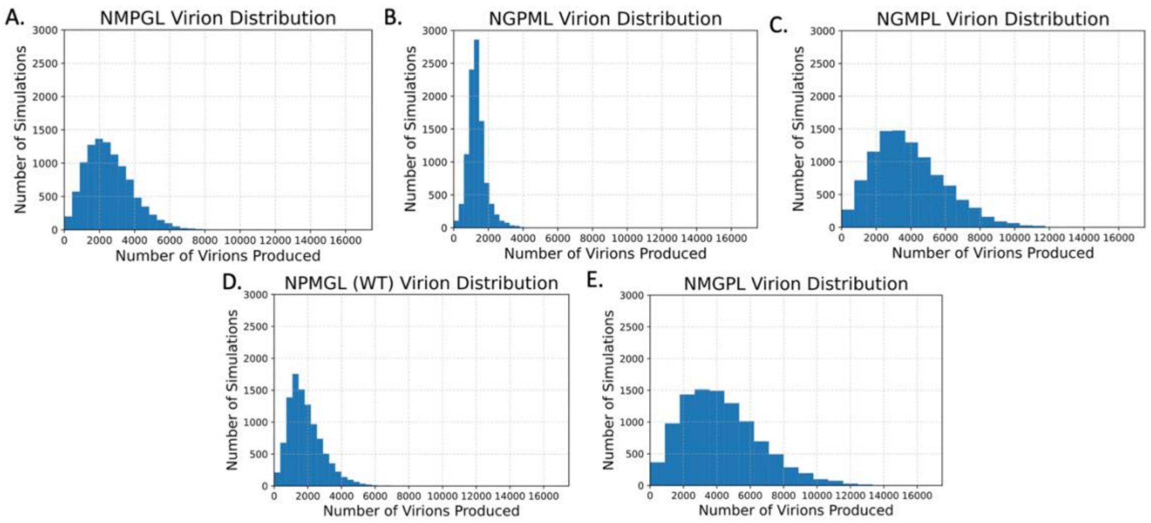

S2 Fig. Distribution of the number of virions produced by each gene shuffled Vesicular Stomatitis Virus variant used to fit the model. Each plot represents 10,000 simulations.
