# Supplementary material for: Stochastic model of vesicular stomatitis virus replication reveals mutational effects on virion production
Source: PLoS Comput Biol. 2024 Feb 7;20(2):e1011373. doi: 10.1371/journal.pcbi.1011373 (PMC10878530; doi:10.1371/journal.pcbi.1011373)
Supplement: S1 Table — (PDF) [file pcbi.1011373.s003.pdf]

S1 Table. Gene Shuffled Vesicular Stomatitis Virus Variants with Associated Statistics

Variant	Mean Virions Produced	Standard Deviation	Percent of Infections Aborted	Mean/Standard Deviation
NMGLP	12365.23	11777.92	0.16	1.05
NGMLP	12355.23	11534.64	0.10	1.07
NMLGP	10742.46	9275.01	0.08	1.16
NLMGP	10673.82	9092.71	0.08	1.17
NGLMP	10629.27	8462.59	0.06	1.26
NLGMP	10481.59	8520.48	0.12	1.23
NMGPL	4294.93	2384.08	0.28	1.80
NGMPL	3914.32	2119.58	0.28	1.85
NMPGL	2516.32	1332.83	0.48	1.89
NPMGL (WT)	1863.50	995.86	0.52	1.87
MNLGP	1670.89	2096.34	1.12	0.80
MNGLP	1661.36	2123.16	0.76	0.78
GNMLP	1627.12	2028.92	0.70	0.80
LNGMP	1619.92	2053.19	0.96	0.79
GNLMP	1611.76	1979.64	0.64	0.81
LNMGP	1609.33	2001.43	0.82	0.80
NGPML	1327.77	544.17	0.32	2.44
MNGPL	1310.00	783.27	0.80	1.67
GNMPL	1205.72	654.81	0.48	1.84
NPGML	1013.45	444.63	0.46	2.28
MNPGL	787.04	406.83	0.68	1.93
GNPML	612.22	259.84	0.62	2.36
PNMGL	491.90	230.82	1.60	2.13
MGNPL	377.70	341.54	2.02	1.11
GMNPL	370.01	332.70	1.78	1.11
PNGML	367.13	145.45	1.02	2.52
MPNGL	270.29	183.96	3.16	1.47
GPNML	229.04	142.79	2.00	1.60
PMNGL	223.40	142.50	3.54	1.57
GLNMP	213.67	365.30	6.30	0.58
NMLPG	212.91	77.97	0.52	2.73

MGNLP	210.50	355.97	6.62	0.59
GMNLP	208.30	332.44	5.92	0.63
LMNGP	206.89	345.25	6.26	0.60
MLNGP	206.72	337.18	6.62	0.61
LGNMP	204.04	339.15	6.74	0.60
NLMPG	192.64	68.35	0.44	2.82
PGNML	192.50	105.70	2.56	1.82
NMPLG	161.26	55.34	0.50	2.91
NLPMG	131.92	45.03	0.46	2.93
NPMLG	109.96	35.92	0.54	3.06
NLGPM	101.14	36.52	0.12	2.77
NGLPM	100.75	37.00	0.16	2.72
NPLMG	99.71	33.72	0.76	2.96
MNLPG	95.37	51.24	1.16	1.86
GMPNL	94.76	101.16	6.60	0.94
MGPNL	92.92	102.26	6.68	0.91
LNMPG	92.57	47.51	0.90	1.95
MPGNL	91.02	89.32	6.78	1.02
GPMNL	88.54	87.78	6.32	1.01
PMGNL	82.81	76.20	7.86	1.09
PGMNL	81.27	72.54	7.20	1.12
NLPGM	77.44	28.14	0.22	2.75
NGPLM	77.33	27.89	0.14	2.77
MNPLG	76.82	38.75	1.70	1.98
LNPMG	68.78	32.47	0.92	2.12
NPLGM	59.61	21.77	0.22	2.74
NPGLM	59.15	22.16	0.44	2.67
LNGPM	49.83	24.26	0.54	2.05
GNLPM	49.62	24.27	0.84	2.04
PNMLG	46.12	21.22	1.80	2.17
PNLMG	41.37	19.01	1.74	2.18
LMNPG	40.37	27.72	2.68	1.46
MLNPG	39.73	28.25	3.34	1.41
GNPLM	39.16	18.82	0.78	2.08
LNPGM	38.99	18.77	0.96	2.08
LGMNP	31.96	63.81	23.12	0.50

MLGNP	30.43	61.71	24.32	0.49
GMLNP	29.75	59.00	24.22	0.50
MPNLG	29.38	18.65	4.72	1.58
LMGNP	29.20	59.28	24.40	0.49
LPNMG	28.91	17.82	3.28	1.62
MGLNP	28.81	55.61	24.68	0.52
GLMNP	28.62	55.44	23.70	0.52
GLNPM	24.69	15.69	3.46	1.57
LGNPM	24.63	15.60	3.40	1.58
PNLGM	24.42	12.29	1.48	1.99
PMNLG	24.05	14.85	5.36	1.62
PNGLM	24.05	12.10	1.50	1.99
PLNMG	23.12	13.85	4.20	1.67
MLPNG	16.99	13.44	8.58	1.26
GPNLM	16.89	10.87	5.00	1.55
LPNGM	16.71	10.62	4.58	1.57
LMPNG	16.47	13.26	8.74	1.24
MPLNG	15.12	11.78	10.22	1.28
LPMNG	14.46	11.29	9.64	1.28
PLNGM	13.22	8.60	6.28	1.54
PGNLM	13.08	8.56	6.40	1.53
PMLNG	12.83	9.74	11.22	1.32
PLMNG	12.74	9.61	10.80	1.33
LGPNM	11.00	8.77	12.42	1.25
GLPNM	10.78	8.88	13.38	1.21
GPLNM	9.33	7.54	14.98	1.24
LPGNM	9.15	7.47	15.10	1.22
PLGNM	7.61	6.38	17.84	1.19
PGLNM	7.56	6.25	18.36	1.21
PLMGN	1.19	1.51	84.28	0.79
PMGLN	1.19	1.51	84.66	0.78
PGMLN	1.16	1.51	85.26	0.76
PMLGN	1.15	1.46	85.08	0.79
PLGMN	1.14	1.45	85.32	0.78
PGLMN	1.12	1.41	85.00	0.79
MPGLN	0.99	1.36	86.68	0.73

LPMGN	0.98	1.37	87.08	0.72
LPGMN	0.97	1.39	86.26	0.70
GPMLN	0.96	1.35	86.90	0.71
MPLGN	0.96	1.36	87.58	0.70
GPLMN	0.95	1.29	86.60	0.73
LMPGN	0.81	1.27	88.58	0.63
LGPMN	0.77	1.16	88.80	0.67
GLPMN	0.77	1.20	89.46	0.64
MGPLN	0.76	1.21	89.86	0.63
MLPGN	0.76	1.20	90.34	0.64
GMPLN	0.74	1.15	90.68	0.65
MGLPN	0.64	1.09	90.62	0.58
LMGPN	0.63	1.07	91.34	0.59
MLGPN	0.62	1.14	91.68	0.54
GMLPN	0.61	1.02	91.04	0.60
GLMPN	0.61	1.09	91.38	0.57
LGMPN	0.60	1.01	91.68	0.59
